# Pharmacological Treatment of Obesity in Older Adults

**DOI:** 10.1007/s40266-024-01150-9

**Published:** 2024-11-08

**Authors:** Ondřej Žižka, Martin Haluzík, Edward B. Jude

**Affiliations:** 1 Diabetes Centre, Institute for Clinical and Experimental Medicine, Prague, Czechia; 2 Department of Diabetes and Endocrinology, Tameside and Glossop Integrated Care NHS Foundation Trust and University of Manchester, Ashton under Lyne, UK; 3 First Faculty of Medicine, Charles University, Prague, Czechia

## Abstract

Obesity is a complex health issue with growing prevalence worldwide. It is also becoming more prevalent in the population of older adults (i.e., 65 years of age and older), affecting frequency and severity as well as other comorbidities, quality of life and consequently, life expectancy. In this article we review currently available data on pharmacotherapy of obesity in the population of older adults and its role in obesity management. Even though there is growing evidence, in particular in the general population, of favourable efficacy and safety profiles of glucagon-like peptide-1 (GLP-1) receptor agonists liraglutide and semaglutide, and recently dual GLP-1 and glucose-dependent insulinotropic polypeptide (GIP) agonist tirzepatide, concise guidelines for older adults are not available to this day. We further discuss specific approaches to frequently represented phenotype of obesity in older adults, in particular sarcopenic obesity and rationale when to treat and how. In older adults with obesity there is a need for more drug trials focusing not only on weight loss, but also on geriatric endpoints including muscle mass preservation, bone quality and favourable fat distribution changes to get enough data for evidence-based recommendation on obesity treatment in this growing sub-population.

## Key Points

**Table:** 

Weight reduction should be considered only in older adults with obesity with related comorbidities after weighing all risks and benefits, keeping in mind possible aggravation of muscle and bone mass loss.
Every weight loss intervention should be accompanied by physical exercise if possible and appropriate protein and macro/micro nutrient intake.
To date there is no official recommendation or guideline for pharmacotherapy of obesity in older adults. From currently available obesity pharmacotherapy, GLP-1 agonist (or combined GLP-1 and GIP agonist) data offer the most promising weight loss, body composition and safety outcomes in older adults; however, more research on muscle/bone mass preservation is needed.

## Introduction

Obesity is a growing global epidemic posing a threat not only to individual health but also to the future stability of health systems due to its increasing prevalence and cost of treatment of obesity-related complications [1].

Obesity is closely associated with increased incidence of metabolic, cardiovascular and other complications including type 2 diabetes, coronary heart disease, chronic heart failure, atrial fibrillation, sleep apnoea syndrome and increased rate of several types of cancer [2–5].

Obesity markedly shortens life span primarily due to excessive cardiovascular mortality and impairs quality of life [6–8].

While obesity-related chronic complications and life expectancy are most significantly affected by weight loss interventions in the middle-aged population, there is also growing prevalence of obesity in older adults. This problem becomes even more pressing with increasing life expectancy in the majority of developed countries worldwide. Obesity-related complications in older people may have even more pronounced effect on quality of life than in the middle-aged, therefore safe and effective treatments of obesity in older adults are urgently needed. One of the significant challenges often present in older adults with obesity is a senescence-associated loss of muscle mass commonly referred to as sarcopenia. As any weight loss includes not only decrease in body fat but also a certain degree of reduction of muscle mass, a complex approach combining dietary intervention, regular multimodal exercise and properly selected weight loss medication is needed in many older adult patients to achieve long-term weight reduction without worsening of sarcopenia. In this article, we focus on currently available data on the use of pharmacological agents for treatment of obesity in the population of older adults.

## Obesity

### Definition and Classification

Currently accepted obesity definition for adult population according to the World Health Organization (WHO) is body mass index (BMI) greater than 30 (kg/m^2^) [9]. BMI is calculated as weight in kilograms divided by double the height in metres. Obesity can be further divided into three sub-classes according to BMI (Table 1), which translate to further increasing disease risk, especially in populations of European ancestry. For Asian populations the cut-off for obesity is lower [10]. Defining obesity and excess fat mass only by BMI calculation has its caveats and limitations, yet it still holds its place as the readiest measurement in the clinical setting.

**Table 1:** Obesity classification

	BMI (kg/m^2^)
Underweight	< 18.5
Normal	18.5–24.9
Overweight	25.0–29.9
Obesity (class 1)	30.0–34.9
Obesity (class 2)	35.0–39.9
Extreme obesity (class 3)	≥ 40

Another clinically available risk predictor is waist circumference measurement used as a part of definition of metabolic syndrome. It takes into account the unfavourable abdominal fat accumulation, which is a standalone disease risk predictor. With ageing, fat preferentially accumulates both viscerally and ectopically rather than as abdominal subcutaneous fat [11], making this measurement even more relevant. Risk thresholds for waist circumference ratio are > 94 cm for men and > 80 cm for women of European ancestry and > 90 cm for men and > 80 cm for women of South Asian, Japanese, and Chinese origin [12]. Original US national guidelines suggest > 102 cm for men and > 88 cm for women [13]. People with predominantly abdominal fat accumulation are considered high-risk even without meeting the BMI obesity criteria [10].

Fat mass can be directly measured by one of several imaging modalities, including underwater weighing, dual-energy x-ray absorptiometry (DXA), computed tomography (CT), magnetic resonance imaging (MRI) and bioimpedance analysis (BIA). Bioimpedance analysis is one of the most clinically available methods to measure body composition, however, it may pose interpretation challenges, especially when there are comorbidities such as congestive heart failure or renal disease present, which are both very prevalent in the population of older adults [10]. DXA is considered the gold standard technique to measure body composition, thanks to its accuracy and low radiation exposure. It is also starting to be frequently used to evaluate muscle mass, which is especially important in older adults, as discussed below [14].

Using BMI as a disease risk predictor in the population of older adults does not take into account the senescence-associated muscle loss and body composition shifts towards increased adiposity or stature decline which can both happen without major changes in BMI. Meta-analysis from Winter et al. [15] including 32 studies with an average follow-up of 12 years revealed that all-cause mortality risk associated with BMI in older adults follows a different trend than in their younger counterparts. All-cause mortality risk associated with BMI was the lowest within the range of BMI 24.0 and 30.9 kg/m^2^ in adults aged 65 years and more. Older adults with overweight (BMI 25.0–29.9 kg/m^2^) had 4–10% lower mortality risk. These findings suggest a different approach to weight maintenance in older adults, advising against major weight loss in and below the overweight range.

The combination of muscle mass loss and increase in adiposity may result in sarcopenic obesity (SO), a difficult-to-manage condition in older population.

### Sarcopenia and Sarcopenic Obesity—a Specific Challenge

Sarcopenia is a progressive and generalised skeletal muscle disorder that is associated with increased likelihood of adverse outcomes including falls, fractures, physical disability and mortality [16].

**Fig. 1:**
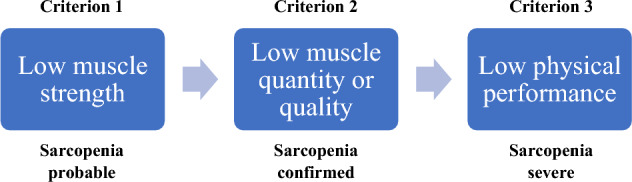
Sarcopenia definition. (Based on criteria from: Sarcopenia: revised European consensus on definition and diagnosis [16].)

The operational definition, updated in 2019 by the European Working Group on Sarcopenia in Older People (EWGSOP2) is outlined in the diagram (Fig. 1) below:

EWGSOP2 recommends the use of the Strength, Assistance, Rise, Climb and Falls (SARC-F) questionnaire as a way to elicit self-reports from patients on signs that are characteristic of sarcopenia (Table 2). The SARC-F is a 5-item questionnaire that is self-reported by patients as a screening for sarcopenia. Responses are based on the patient’s perception of their limitations in strength, walking ability, rising from a chair, stair climbing and experiences with falls.

**Table 2:** SARC-F questionnaire [17]

Component		Question	Scoring
Strength		How much difficulty do you have in lifting and carrying 10 pounds?	None, 0 Some, 1 A lot or unable, 2
Assistance in walking		How much difficulty do you have walking across a room?	None, 0 Some, 1 A lot, use aids or unable, 2
Rise from a chair		How much difficulty do you have transferring from a chair or bed?	None, 0 Some, 1 A lot or unable without help, 2
Climb stairs		How much difficulty do you have climbing a flight of 10 stairs?	None, 0 Some, 1 A lot or unable, 2
Falls		How many times have you fallen in the past year?	None, 0 1–3 falls, 1 4 or more falls, 2

To assess sarcopenia, the working group recommends grip strength and chair stand test for muscle strength, short physical performance battery (SPPB), timed-up and go (TUG) test and 400-m walk test for physical performance. Furthermore, DXA, MRI and CT imaging can be used to determine muscle quantity, more precisely skeletal muscle mass (SMM), as appendicular skeletal muscle mass (ASM). BIA can be also used for estimation of total skeletal muscle with limitations mentioned above. Detailed information about the individual tests alongside with optimal thresholds can be found in the Consensus [16].

Sarcopenic obesity is defined as the co-existence of obesity and sarcopenia [18].

Until recently, there was no unified definition of sarcopenic obesity, thus affecting variation of prevalence data which could vary 26-fold depending on research criteria as reported by Batsis et.al. [19]. Consensus statement by the European Society for Clinical Nutrition and Metabolism (ESPEN) and European Association for the Study of Obesity (EASO) from 2022 defines sarcopenic obesity as the co-existence of obesity defined by high body fat, and sarcopenia defined by low skeletal muscle mass in combination with low muscle function. The Consensus recommend a three-step diagnostic approach of screening, diagnosis and staging. Screening for SO is focussed on elevated BMI or waist circumference together with risk factors and clinical symptoms of sarcopenia, also using validated questionnaires such as SARC-F questionnaire mentioned previously. Diagnosis is made from altered skeletal muscle functional parameters reflecting muscle strength, more precisely hand-grip strength (HGS), knee extensor strength or chair-stand tests with validation of cut-off points according to sex, ethnicity and age [18]. The second step in diagnosis is body composition assessment by DXA, or BIA as an alternative second choice. CT should be used when possible [18], for example, in patients already undertaking the examination for other diagnosis. Staging of SO is based on presence of one or more complications attributable to body composition and skeletal muscle function alterations, for example, metabolic diseases and disabilities (stage II), or absence thereof (stage I) [18].

Sarcopenic obesity is associated with increased risk of disability, metabolic impairments (i.e. dyslipidaemia and insulin resistance) and comorbidities such as type 2 diabetes mellitus or osteoarthritis [11]. Low handgrip strength and elevated BMI were strongly associated with an increased risk of functional decline compared with obesity and dynapenia alone [20]. Weight loss may pose a risk of aggravation of sarcopenia in individuals with sarcopenic obesity, as seen in caloric restriction (CR) approach alone. Meta-analysis [21] of 33 intervention studies lasting 8–24 weeks reported that calorie restriction alone without resistance training leads to the loss of muscle mass and loss of handgrip strength [11, 21]. Hypocaloric diet therapy without exercise in older frail adults ≥ 65 years with obesity (BMI ≥ 30 kg/m^2^) led to a marked loss of lean mass at 1 year (3.2 ± 2.0 kg, 5% change from baseline) compared with the diet and exercise group, where the loss of lean mass was partially mitigated (decrease of 1.8 ± 1.7 kg, 3% change from baseline); (*p* = 0.04) [22]. This phenomenon should be considered in individuals with sarcopenic obesity and may pose a challenge in other treatment modalities focussing on decreasing adiposity by restricting calorie intake both directly and indirectly.

### Obesity Paradox in Older Adult Patients

Obesity paradox is a term that often comes up when discussing treatment of obesity in the population of older adults as perhaps an argument against excessive weight loss. Obesity paradox has been described as a “paradoxical” association of higher BMI as a protective factor in clinical outcomes of chronic diseases such those of cardiovascular system, hypertension and atrial fibrillation. This “paradox” has also been described in other conditions such as cancers, chronic kidney disease, chronic obstructive pulmonary disease, stroke, pulmonary hypertension, osteoporosis, critical illness and sepsis [23]. Among other possible mechanisms, the cardioprotective role of adipokines was proposed as in individuals with normal weight or overweight, where increased leptin levels without developed leptin resistance were associated with better cardiovascular outcomes (i.e., cardiovascular death, myocardial infarction, stroke). In contrast, leptin resistance in obesity characterized by increased leptin/adiponectin ratio diminishes favourable cardiovascular outcomes in these patients [24]. The precise role of adipokines and their effect on possible obesity paradox needs to be examined further.

Some studies also suggest that sarcopenic obesity compared with sarcopenia alone might be associated with lesser risk of frailty and better performance in functional tests such as TUG test or instrumental activities of daily living (IADL), more research and clarification is, however, needed [25, 26].

The narrative overview performed by Bosello et al. [27] concluded that the phenomenon of obesity paradox may be often inaccurately assessed in studies, underestimating harmful impacts of obesity on morbidity and mortality without considering metrics such as body composition, muscle mass, fat distribution and other confounding factors. This is in accordance with finding from Simati et al., mentioning limitations of BMI use in evaluation of this phenomenon [23]. More research is therefore necessary, especially in the population of older adults, often suffering from concurrence of many chronic diseases.

### Epidemiology and Future Predictions

As discussed before, obesity is a worldwide growing health issue, nearly tripling in prevalence from 1975 to 2016 [28].

In evaluation performed by the WHO in 2016, more than 1.9 billion adults worldwide aged 18 years and older were overweight, of which 650 million were obese, which makes up 39% of adults aged 18 years and older being overweight and 13% obese. Overweight and obesity are linked to more deaths worldwide than underweight. Globally there are more people who are obese than underweight except in some regions of Asia and Africa [28].

Prevalence of obesity by age in the USA was examined by the Centers for Disease Control and Prevention (CDC) between the years 2015 and 2018, with prevalence of obesity in the age group of 65–74 years being 45.9% for women and 41.9% for men as compared with prevalence of 46.2% in the age group 55–64 years and 43.1% in 45–54 years for women, 43.9% in the age group 55–64 years and 42.9% in 45–54 years for men, respectively. In the group 75 years and older, the prevalence was 36.1% for women and 31.8 % for men, respectively. There was also significant difference in prevalence of obesity in adults regardless of age between different race groups, in people of Black or African American origin 48.6%, Hispanic or Latino origin 45.7%, white 40.2% and Asian 14.9% [29].

In the Health Survey for England 2021, prevalence rates of obesity were 32% in the age group 65–74 years and 26% in the age group 75 + years, respectively [30].

Prevalence of obesity in population of older adults varies depending on the region, however, more complex age-specific worldwide statistic data are missing. Future projections suggest a much larger proportional increase in the number of overweight and obese individuals by 2030 in developing nations compared with developed regions as a result of sociodemographic changes, less accessible dietary education and poorer health literacy [31].

Another factor that comes into play is the ageing of population. By 2050, the world’s population of people aged 60 years and older is estimated to double to 2.1 billion [32], of which 80% will be living in low- and middle-income countries. This fact combined with growing trend of obesity and its complications poses a great challenge on future organisation and sustainability of healthcare systems worldwide.

## Management of Obesity in Older Adults

Indication for weight management intervention in older adults should be considered carefully and individually. As discussed previously, the risk arising from further decrease of muscle mass and progression of frailty is present with almost every weight-reducing intervention. Current clinical consensus and guidelines advise against weight reduction in older adults with overweight to prevent loss of muscle mass and functional decline. Instead, it is recommended for overweight older adults to maintain stable body weight to avoid progression to both obesity or sarcopenic obesity [33].

Older adults with obesity and related health complications (i.e. metabolic, cardiovascular, orthopaedic) should be selected for weight loss intervention only after careful individual weighing of risks, benefits and patient’s priorities. Physical exercise should accompany every weight-reducing intervention if possible alongside with proper nutritional support [33].

### Nutritional Intervention and Physical Activity

Nutritional intervention and physical activity should always be in the forefront of every obesity treatment strategy regardless of age. Moderate weight loss (up to 10% of baseline weight over 3–12 months) in conjunction with physical activity may improve physical function, metabolic outcomes and reduce cardiovascular risks. This is, however, often accompanied by reduction of skeletal muscle mass and bone mineral density, possibly worsening sarcopenia further [34]. This effect can be attenuated but not eliminated completely by concomitant exercise [22].

Only mild calorie deficit (approximately 500 kcal/day) from the daily energy requirement is recommended while maintaining a minimum caloric intake of 1000–1200 kcal/day for a sustainable weight loss of 0.25–1 kg/week [33]. Nutritional intervention should also include enough protein intake (1–1.5 g/kg body weight (BW)/day), calcium intake (1000–1200 mg/day) accompanied by micronutrient supplementation, that is, magnesium, vitamin B6, vitamin B12 and selenium [35]. Vitamin D is needed to mitigate unfavourable effect of weight loss on bone mineral density; some evidence also mentions beneficial effect of vitamin D supplementation on muscle strength and function in patients with sarcopenia [11, 36]. Supplementation of essential amino acids, more specifically leucine (minimum 2 g/day) or its metabolite β-hydroxy-β-methylbutyrate (HMB, also 2–3 g/day) is proposed as well [35].

Physical activity should combine strength, endurance, flexibility and balance exercises in older adults. American College of Sports Medicine (ACSM) recommends weekly a minimum of 150 minutes of moderate-intensity aerobic activity or 75 minutes of vigorous-intensity aerobic activity. In addition, two or more days of moderate-intensity strengthening activities, with 8–10 exercises focussing on major muscle groups, is recommended. Patients should strive to perform 2 hours of balance exercises per week and low intensity stretching daily [37]. Training plan of three times a week 90 minute sessions, consisting of 15 minutes of balance training, 15 minutes of flexibility training, 30 minutes of aerobic exercise and 30 minutes of high-intensity resistance training has also been proposed for older adults [35].

**Fig. 2:**
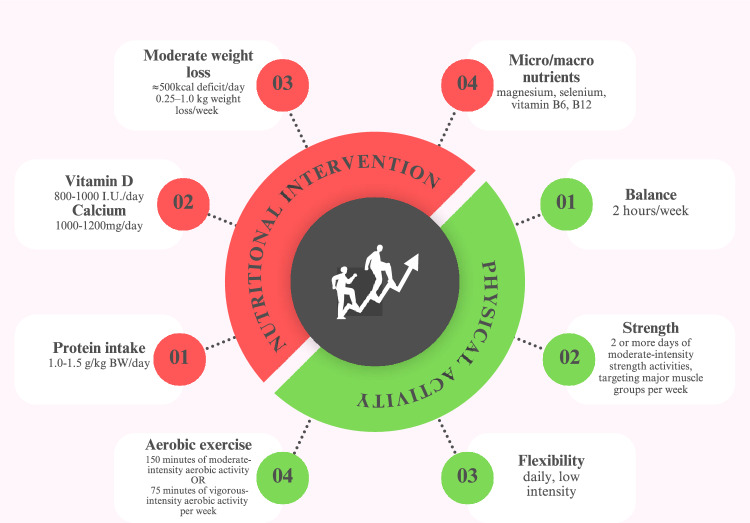
Lifestyle interventions for treatment of obesity in older adults

Recommended lifestyle interventions for treatment of obesity in older adults are summarized in Fig. 2.

### Bariatric Surgery

Bariatric surgery is the most effective treatment of obesity [38] and type 2 diabetes [39]. Sleeve gastrectomy (SG) and Roux-en-Y gastric bypass (RYGB) are the most commonly used bariatric procedures worldwide [40].

Despite a similar prevalence of obesity compared with younger age groups, patients older than 65 years of age represent less than 7% of the population undergoing bariatric surgery. This is at least in part due to the fact that recommended age for bariatric surgery was traditionally 18–60 years with a possibility to operate on older patients on an individual basis when benefits outweigh the risks [41].

In a cohort study performed by Iranmanesh et al., significant weight loss and comorbidity improvement were achieved in both groups of 22,981 patients < 65 years of age and 532 patients ≥ 65 years. Overall postoperative complications were similar between patients < 65 and ≥ 65 years 3388/22,981 (14.7%) versus 73/532 (13.7%, *p* = 0.537) as well [41].

There is, however, mounting evidence that bariatric surgery in older adult patients could be associated with increased in-hospital mortality, complications after surgery and consequently greater healthcare costs. Retrospective study by Mabeza et al. included 351,292 patients undertaking bariatric surgery (elective laparoscopic gastric bypass or sleeve gastrectomy), of which 44,183 (12.6%) belonged to geriatric cohort. This study revealed that patients aged 65 years and more had higher unadjusted rates of in-hospital mortality (0.3% versus 0.04% in non-older adult patients, *p* < 0.001), and complications, that is, cardiac (0.4 versus 0.2%, *p* < 0.001), respiratory (1.2 versus 0.5%, *p* < 0.001), renal (2.6 versus 1.0%, *p* < 0.001) and thromboembolic (0.2 versus 0.1%, *p* < 0.001) compared with their younger counterparts. Older adults also experienced prolonged hospitalization more often and had higher associated hospitalization costs [42].

This finding supports the need for careful weighing of risks versus benefit including geriatric assessment while deciding the appropriate treatment modality in older adults. Bariatric surgery is one of the promising strategies in treatment of obesity. More detailed discussion of this treatment modality in older adult individuals is, however, beyond the scope of this publication.

### Pharmacotherapy

Pharmacotherapy is, in the last couple of years, the fastest developing and promising treatment strategy for obesity and related complications. However, available data focusing on the older adult population, that is, age > 65 years, are still very scarce. Representation of this age group in clinical trials is often very limited or not evaluated separately and more closely or it is excluded completely. Trials evaluating currently available obesity medications often focus mainly on weight reduction endpoints, which is not always the main goal from a geriatric viewpoint. This is especially true when the weight loss is accompanied by significant lean muscle mass reduction, which may aggravate pre-existing sarcopenia and result in frailty with related functional limitations. Other relevant geriatric outcomes such as muscle strength and bone mineral density are usually not part of the endpoint evaluation. We have selected currently approved pharmacotherapy for obesity and reviewed data for its use in the older adult population.

#### GLP1 Receptor Agonists

Glucagon-like peptide 1 (GLP-1) receptor agonists were initially used as a therapy for type 2 diabetes, thanks to their incretin role in glucose metabolism. It has been proven that systemic administration of GLP-1 or GLP-1 receptor agonists, in addition to improving glucose control decreases food intake, slows gastric emptying and reduces body weight by central anorexigenic effect [43, 44].

##### Liraglutide

Liraglutide in a maximum dose of 1.8 mg once daily as a subcutaneous injection has been used for the treatment of diabetes since 2009 [45]. It was also approved in 2014 by the Food and Drug Administration (FDA) and in 2015 by the European Medicines Agency (EMA) as a treatment for obesity with maximum dose of 3 mg once daily. It is indicated as an adjunct to a hypocaloric diet and increased physical activity for weight management in adult patients with obesity (BMI of ≥ 30 kg/m^2^), or overweight (BMI ≥ 27 kg/m^2^ to < 30 kg/m^2^) in the presence of at least one weight-related comorbidity such as prediabetes or type 2 diabetes mellitus, hypertension, dyslipidaemia or obstructive sleep apnoea in adults. Treatment with liraglutide should be discontinued after 12 weeks on the 3.0 mg/day dose if patients have not lost at least 5% of their baseline body weight [46].

The starting dose of liraglutide is 0.6 mg once daily. The dose should be increased to 3.0 mg once daily in increments of 0.6 mg with at least 1-week intervals to prevent/reduce gastrointestinal side effects. Doses higher than 3.0 mg are not recommended. No dose adjustment is necessary in older adults aged 65 years and more, however, therapeutic experience in population in patients ≥ 75 years of age is limited and use in these patients is not recommended due to lack of data [46].

Liraglutide clinical safety and efficacy was evaluated in the SCALE trials. The original SCALE Obesity and Pre-Diabetes trial resulted in a total of 63.2% of the patients in the liraglutide group as compared with 27.1% in the placebo group losing at least 5% of their body weight (*p* < 0.001), and 33.1% versus 10.6% losing more than 10% of their body weight (*p* < 0.001) [47]. In SCALE Obesity and Prediabetes and SCALE Diabetes randomized control trials, once daily liraglutide 3.0 mg administration, as an adjunct to diet and exercise, showed similar mean and categorical weight loss efficacy in individuals aged ≥ 65 and < 65 years. In both trials, the proportion of individuals reporting adverse events tended to increase with age for each treatment. In SCALE Obesity and Prediabetes trial the serious adverse event rate was 6.1% versus 4.5% for liraglutide 3.0 mg compared with placebo in the < 65 years of age subgroup and 8.8% versus 13.2 % in the ≥ 65 years of age subgroup. In SCALE Diabetes trial the serious adverse event rate was 7.7% versus 5.7% for liraglutide 3.0 mg versus placebo in the < 65 years of age subgroup and 12.9% versus 7.9% in the ≥ 65 years of age subgroup. Hepatobiliary disorders were more common in the < 65 years of age subgroup, gastrointestinal events were more frequent with liraglutide 3.0 mg than placebo in both groups and benign, malignant and unspecified neoplasms were more common in the ≥ 65 years age subgroup [48].

A small trial by Perna et al. demonstrated that 24 weeks treatment with 3.0 mg of liraglutide was associated with reduction of fat mass and preserved skeletal muscle mass in nine individuals with both overweight or obesity and type 2 diabetes with mean age of 68.22 ± 3.86 years with baseline lack of physical activity and sedentary lifestyle [49]. The median fat mass reduction was − 1498 g and preserved skeletal muscle mass index (SMI) of + 0.03 kg/m^2^ median. Importantly, none of the tested subjects progressed into sarcopenia; in one individual the treatment showed anabolic effect with augmentation of fat free mass by + 1134 g. No protein supplementation or enhancement of physical activity was part of the intervention. Physical activity was assessed by physical activity scale for the elderly (PASE) questionnaire, with median change of − 11.60 points at 24 weeks. The recent data show, however, that when assessing skeletal muscle mass in obese and overweight individuals, adjustment of muscle mass to BMI or body weight is more accurate in revealing sarcopenia compared with adjustment to height^2^, which might underdiagnose sarcopenia in obese individuals [50]. This fact might be a confounding factor in this or similar studies.

Possible muscle preserving effect as mentioned above is in accordance with findings of Xiang et al., who examined effect of GLP-1 agonists liraglutide and semaglutide on muscle mass and muscular atrophy in murine models of obesity. In this study both GLP1-RA attenuated fat mass gain induced by high-fat diet (HFD) fed mice and preserved muscle strength normally reduced by HFD via activation of sirtuin-1 associated pathways, which is a key player in the ageing processes as well [51].

Liraglutide was also evaluated in terms of efficacy and safety in older adult patients with type 2 diabetes with insulin therapy [52]. The treatment with doses up to 0.9 mg after for 6 months in combination with insulin regimen improved blood glucose variability HbA1c levels and decreased insulin doses and body weight (60.0; 55.4–72.8 kg baseline to 56.7; 52.2–70.5 kg, *p* = 0.006, post-treatment). The safety profile of liraglutide remained favourable throughout the trial, with only 2 of 20 participants experiencing nausea or diarrhoea, and were able to continue after liraglutide dose reduction. No other adverse effects were reported.

Effect of liraglutide on cardiovascular events was favourable in patients aged both 60–74 and 75 + years, respectively, with type 2 diabetes at high risk of cardiovascular disease, showing reduction of major adverse cardiovascular events (MACEs) by 13% in the entire population of the LEADER trial [53]. Patients aged 75 years or older had a 34% and 29% risk reduction in the frequency of MACEs and expanded MACE outcomes, respectively, comparing liraglutide versus placebo. The risk reduction in all-cause mortality with liraglutide versus placebo was 35% in patients aged 75 years or older versus 6% in patients aged 60–74 years showing interesting advantages in the older age group. Overall, 63.5% and 61.7% of patients aged 75 years or older reported serious adverse events and non-serious medical events of special interest, respectively, versus 49.5% and 49.8% of patients aged 60–74 years. The most common adverse events were neoplasms (10.3% in patients aged 60–74 years compared with 14.2% patients 75 years or older) and gastrointestinal disorders (diarrhoea, nausea and vomiting in 1.2%, 2.4% and 1.1% of patients aged 60–74 years and 2.8%, 2.9% and 1.2% in those aged 75 years or older, respectively). In the liraglutide group versus the placebo group, more patients had gastrointestinal adverse events (46.6% versus 33.0%) and the incidence of acute gallstone disease was higher (10.0% versus 6.3%), regardless of age. The overall benefits seemed more pronounced in the older age group including the influence on cardiovascular outcomes. Obesity, weight loss and muscle preservation were not among the evaluated endpoints of this post hoc analysis [53].

There is also growing evidence that liraglutide may have a positive effect on progression of dementia in older adult patients with type 2 diabetes [54]. Recently, beneficial effect of liraglutide on impaired associative learning in individuals with obesity was also reported by Hanssen et al. [55]. More thorough research is necessary, however, a positive effect on cognition would be another argument for use of liraglutide in obese older adult population.

Liraglutide is effective medication for weight loss with years of clinical practice and robust evidence. More data on muscle mass preservation is necessary as with other weight reducing drugs. Adverse effects (mainly gastrointestinal) tend to be more pronounced in older population, increasing risk of dehydration, malnutrition and consequently, sarcopenia and frailty and should be kept in mind when prescribing liraglutide.

##### Semaglutide

Semaglutide is another GLP-1-receptor agonist approved for use in patients with type 2 diabetes and/or obesity.

OZEMPIC (injectable form) and RYBELSUS (oral form) were both approved by EMA and FDA for the treatment of type 2 diabetes mellitus in adults as an adjunct to hypocaloric diet and exercise in monotherapy when metformin is considered inappropriate or in addition to other medications for the treatment of diabetes. Semaglutide is licenced for 1 mg once-weekly subcutaneous injection or in dose of 14 mg once daily in the oral form [56].

WEGOVY (injectable form), on the contrary, is indicated as an adjunct to a hypocaloric diet and increased physical activity for weight management, including weight loss and weight maintenance, in adults with BMI of ≥ 30 kg/m^2^ (obesity) or ≥ 27 kg/m^2^ to < 30 kg/m^2^ (overweight) in the presence of at least one weight-related comorbidity, for example, prediabetes or type 2 diabetes mellitus, hypertension, dyslipidaemia, obstructive sleep apnoea or cardiovascular disease [56].

The therapy is escalated over 16-week period to reach the maximum maintenance dose of 2.4 mg of subcutaneous semaglutide once weekly, starting with a dose of 0.25 mg once weekly to reduce the risk of gastrointestinal adverse events. The therapy escalation should be delayed if there are significant gastrointestinal symptoms present, until their subsidence. Weekly doses higher than 2.4 mg are currently not recommended, however, there is an ongoing trial testing the doses up to 7.2 mg once weekly. No dose adjustment is recommended in the older adult population, however, data in patients 75 years of age and older are limited [56].

Efficacy and safety of once-weekly semaglutide was evaluated in the SUSTAIN series trials in which adults with type 2 diabetes were randomized to semaglutide 0.5 mg and semaglutide 1.0 mg and comparator (placebo, sitagliptin, insulin glargine, other GLP-1 agonists) medical treatments with primary endpoint being reduction of glycated haemoglobin and secondary endpoint being weight loss. The post hoc analysis [57] of SUSTAIN 1–5 trials included 854 (21.9% of total) older adult subjects with mean age of 69–70 years. Semaglutide reduced bodyweight comparably between non-older adult and older adult age group, with 0.5 mg semaglutide weekly, body weight reductions ranged from − 3.3 to − 4.3 kg in non-older adult patients and − 3.6 to − 4.6 kg in older adult patients, and from − 4.6 to − 6.4 kg in non-older adults and − 4.1 to − 6.7 kg in older adults with semaglutide 1.0 mg weekly. Glycated haemoglobin reduction was also comparable in both groups [58].

Adverse events were compared as well, with severity of AE being similar in both age groups, more patients from the older adult group reported gastrointestinal adverse events. More older adult subjects discontinued the treatment prematurely compared with non-older adult group due to adverse events [57].

In the SUSTAIN 7 trial, semaglutide and dulaglutide were compared head-to-head in subjects on background treatment with metformin. Interestingly, proportion of older adult versus non-older adult subjects achieving glycaemic targets and weight loss response of ≥ 5% was consistently higher with both semaglutide and dulaglutide despite older adult subjects having a lower baseline HbA1c and BMI than non-older adult subjects. Proportions of subjects achieving ≥ 10% weight loss were comparable between the two age subgroups for both treatment arms [59]. However, evaluation of body weight reduction did not take into account body composition changes, that is, fat mass and its distribution versus lean mass decrease, an important factor in evaluating obesity treatment in the older adult population. Adverse events were reported in more than a half of subjects in SUSTAIN 7 trial regardless of age category, with gastrointestinal AE being the most frequent issue.

Once-weekly injectable semaglutide was also evaluated as a weight loss medication in the series of STEP randomized controlled trials. STEP 1 trial showed 14.9% reduction in body weight during 68 weeks of treatment with 2.4 mg of semaglutide combined with lifestyle intervention compared with 2.4% body weight reduction in the placebo plus lifestyle intervention group [60]. A subgroup of 140 participants underwent a DXA measurement, with result being a total fat mass and regional visceral mass reduction from baseline with treatment with semaglutide. Total lean body mass also decreased in absolute terms; the proportion of lean body mass relative to total body mass increased after treatment with semaglutide [60]. STEP 8 trial also showed superior bodyweight reduction in patients treated with once-weekly 2.4 mg semaglutide compared with daily 3.0 mg liraglutide. At week 68, the estimated mean change in body weight was – 15.8% with semaglutide and – 6.4% with liraglutide [61]. The differences between older adult and non-older adult populations, however, were not part of STEP trials analyses, as age-group-specific data were not evaluated separately.

Once-daily oral semaglutide as a glucose-lowering medication was evaluated in series of PIONEER trials. A subgroup analysis of the PIONEER 9 and 10 Japan trials showed dose-dependent body weight reduction without consistent pattern in both age groups of < 65 years of age and > 65 years of age. Adverse events occurred in larger proportions of older adult patients compared with the non-older adult population. The frequency of serious AEs was low in patients receiving oral semaglutide in PIONEER 9 and 10 and was similar in both age groups. Gastrointestinal adverse events were the most common AEs in both groups [62]. Body composition changes were not a part of the PIONEER trial evaluation, similarly to the STEP or SUSTAIN trials.

Muscle-preserving and at the same time fat-reducing properties of oral semaglutide were observed in 25 patients with type 2 diabetes aged 20–78 years (54.1 ± 2.7, mean ± standard error) in a study performed by Uchiyama et al., who used bioimpedance electrical analysis (BIA) to analyse body composition [63]. This 24-week trial by an individually adjusted dose by tolerability (3.0 mg minimal dose at 24 weeks, 14 mg maximal dose at 24 weeks) of oral semaglutide was accompanied by a decrease of both BMI and whole-body fat. BMI was 29.3 ± 0.68 kg/m^2^ at baseline, 28.5 ± 0.72 kg/m^2^ at 12 weeks (*p* < 0.01) and 28.0 ± 0.71 kg/m^2^ at 24 weeks (*p* < 0.001 versus baseline). Whole-body fat decreased from 28.3 ± 1.52 kg at baseline to 26.8 ± 1.59 kg at 12 weeks (*p* < 0.01) and 25.5 ± 1.57 kg at 24 weeks (*p* < 0.001 versus baseline). Muscle mass preservation was estimated by whole-body lean mass and skeletal muscle index (SMI), which was defined as appendicular skeletal muscle mass adjusted for height^2^. Baseline muscle mass was 48.1 ± 1.92 kg, 47.7 ± 1.93 at 12 weeks and 47.6 ± 1.89 kg at 24 weeks. SMI was 8.1 ± 0.20 kg/m^2^ at baseline, 8.1 ± 0.20 kg/m^2^ at 12 weeks and 8.1 ± 0.20 kg/m^2^ at 24 weeks. There was no subgroup analysis of older adult participants, however, the authors did not observe any significant correlations between age and changes in body composition at 24 weeks, suggesting effects of semaglutide being independent on age [63]. As discussed previously, using skeletal muscle mass adjusted for body weight or BMI would be more appropriate to assess muscle mass preservation in obese individuals.

Semaglutide is a promising effective agent in the treatment of obesity in both non-older adult and older adult populations, however, the effect on muscle mass preservation needs to be evaluated further to assure its position in treatment of older adults with sarcopenic obesity. The age group of subjects aged 75 years and older were also poorly represented in all performed trials, which has to be taken into account when deciding about safety for older adult population. As with other GLP-1 agonists, gastrointestinal adverse effects (i.e. nausea, diarrhoea, constipation) should be considered in older adult population as it may aggravate pre-existing health conditions, including sarcopenia.

##### Tirzepatide

Tirzepatide is a dual glucose-dependent insulinotropic polypeptide (GIP) and GLP-1 agonist. It has a comparable GIP receptor binding affinity to native GIP and five times lower GLP-1 receptor affinity than that of native GLP-1 [64].

Tirzepatide (MOUNJARO) was approved in 2022 by both EMA and FDA for treatment of type 2 diabetes mellitus, and recently, in November 2023, FDA approved and EMA recommended change of the marketing authorisation for the use of tirzepatide for treatment of obesity and overweight in adults with BMI > 27kg/m^2^ or greater with presence of comorbidities, similarly to liraglutide and subcutaneous semaglutide [65, 66].

Tirzepatide is administered subcutaneously once weekly with a starting dose of 2.5 mg with escalation over 4–20 weeks to achieve the target dosage of 5.0 mg, 10.0 mg and 15.0 mg. In contrast to semaglutide where higher doses are used for the weight loss indication, the dosing of tirzepatide is the same for antidiabetic and weight loss indications [65].

In ZEPBOUND clinical trials (tirzepatide for treatment of obesity), 226 (9%) tirzepatide-treated patients were 65 years of age or older, and 13 (0.5%) tirzepatide-treated patients were 75 years of age or older at baseline; no overall differences in safety or effectiveness have been observed between patients > 65 years of age and younger patients. Similarly, in the pool of seven MOUNJARO clinical trials, 1539 (30.1%) patients treated with tirzepatide were 65 years of age or older, and 212 (4.1%) tirzepatide-treated patients were 75 years of age or older at baseline. No significant differences in efficacy and safety were observed across different age groups [67]. This is in accordance with recommendations of EMA for tirzepatide, which recommends no dose adjustment on the basis of age, sex, race, ethnicity or body weight [68]. It is important to mention that older adult participants of these studies probably do not reflect the heterogenous older adult population with all possible comorbidities and complications. Exclusion criteria often include prior cardiovascular, endocrine, renal and hepatogastric disorders which are very prevalent in the older adult population, favouring healthier individuals.

Tirzepatide was evaluated in the series of SURPASS trials, where effect on diabetes compensation and weight loss was thus far most effective of the available glucose lowering agents. SURPASS 1 trial showed significant weight reduction at 40 weeks. Body weight reduction was progressive and dose dependent, − 7.0 kg with tirzepatide 5 mg, − 7.8 kg with tirzepatide 10 mg and − 9.5 kg with tirzepatide 15 mg, versus − 0.7 kg in the placebo group. A greater proportion of participants had bodyweight reductions of 5% or greater (67–78%), 10% or greater (31–47%) and 15% or greater (13–27%) with tirzepatide versus 14%, 1% and 0% with placebo. BMI and waist circumference were reduced significantly as well [69]. The marked effect was also seen on diabetes compensation, with 87–92% of participants reaching HbA1c concentration of less than 7.0% (< 53 mmol/mol) after treatment with tirzepatide compared with 19% with placebo [69].

Pooled post hoc analysis of SURPASS 1-5 trials compared effects of treatment with tirzepatide in patients aged 60 years and more with their younger counterparts. In both age groups, tirzepatide reduced body weight versus comparator group. With tirzepatide 5 mg, the reduction in body weight ranged from ≥ – 7 kg to ≤ – 7.6 kg in the younger group and ≥ – 5.4 kg to ≤ – 7.1 kg in the older adult group. Similarly, tirzepatide 10 mg reduced weight from ≥ – 7.8 kg to ≤ – 10.7 kg in the younger group and from ≥ – 7.5 kg to ≤ – 9.5 kg in the older adult group, and tirzepatide 15 mg from ≥ – 9.5 kg to ≤ 12.9 kg in the younger group and ≥ – 8.8 kg to ≤ – 11.7 kg in the older adult group. In comparison, the comparator group lost from ≥ – 0.7 kg to ≤ + 2.3 kg of body weight. The baseline age for all tirzepatide doses had no significant effect on body weight reduction effect (for tirzepatide 5 mg: *p* = 0.198, tirzepatide 10 mg: *p* = 0.600 and tirzepatide 15 mg: *p* = 0.608) [70]. Unfortunately, this post hoc analysis did not include age specific adverse event reports or body composition assessment before and after intervention, both very important in the older adult group.

The most frequent adverse events with tirzepatide in SURPASS trials were mild-to-moderate gastrointestinal events, including nausea (12–18% versus 6%), diarrhoea (12–14% versus 8%), and vomiting (2–6% versus 2%) compared with participants who received placebo. Most reports of nausea, vomiting, and diarrhoea were mild to moderate in severity and decreased over time in all groups [69].

Tirzepatide was and still is being evaluated in people with obesity (BMI 30 kg/m^2^ or more, or a BMI of 27 kg/m^2^ or more and at least one weight-related complication (e.g. hypertension, dyslipidaemia, obstructive sleep apnoea or cardiovascular disease) in the SURMOUNT trials. In the SURMOUNT-1 trial including patients with obesity (BMI > 30 kg/m^2^ , or BMI > 27 kg/m^2^ and at least one weight-related complication), the mean change in body weight at week 72 was − 15.0 % (95% CI − 15.9 to − 14.2%) with a 5.0 mg weekly dose of tirzepatide, − 19.5% (95% CI − 20.4 to − 18.5%) with a 10.0 mg dose and − 20.9% (95% CI − 21.8 to − 19.9%) with a 15.0 mg dose and − 3.1% (95% CI − 4.3 to − 1.9%) with placebo, respectively. SURMOUNT-1 evaluation also included a subgroup of 160 participants who underwent DXA to analyse body composition changes with mean reduction in total body fat mass of 33.9% with tirzepatide, compared with 8.2% with placebo. The ratio of total fat mass to total lean mass decreased more with tirzepatide (from 0.93 at baseline to 0.70 at week 72) compared with placebo (from 0.95 to 0.88) [71]. Detailed and weight/BMI adjusted lean muscle mass data would provide more accurate insight into the effect of tirzepatide on muscle mass preservation, however, they were not part of the original evaluation.

An age group post hoc analysis of SURMOUNT-1 trial [72] evaluated effect on body composition changes in subpopulations undertaking DXA scan within age subgroups under 50 years (*n* = 99), 50–64.9 years (*n* = 41) and 65 years and older (*n* = 20). Fat mass was reduced 33–36% and lean mass 10–11% depending upon age group, which resulted in improvement of body composition. Across all the age groups, the change was almost identical, indicating no evidence of excess lean mass loss in older age groups [72].

Overall, 78.9–81.8% of participants treated with tirzepatide reported at least one adverse event during the treatment compared with 72.0% of participants in the placebo group. The most frequently reported adverse events were gastrointestinal (nausea, diarrhoea and constipation). Serious adverse events were reported by 6.3% of participants, however, approximately 21% of these serious adverse events were considered to be attributable to coronavirus disease 2019 (COVID-19) infection, which affected patients in all treatment groups.

Safety and efficacy were also evaluated across age and BMI subgroups in East-Asia SURPASS programme. Compared with populations of European descent, East Asian patients with diabetes tend to present with lower BMI, but proportionally higher abdominal adiposity, and at any given BMI. In this trial, participants were treated with tirzepatide 5.0 mg, 10.0 mg or 15.0 mg and evaluated to assess the safety and efficacy of tirzepatide in people of East Asian descent (94% from Japan) on the basis of age (< 65 and ≥ 65 years) and BMI (< 25 and ≥ 25 kg/m^2^). At week 52, tirzepatide induced a similar dose-dependent reduction in glycated haemoglobin, waist circumference and BMI across all sub-groups, with similar safety profile in both age groups; only treatment discontinuation was higher in the group 65 years of age and older due to adverse events [73].

Tirzepatide is a very effective anti-obesity drug with great weight reduction effect and acceptable safety profile, however, in the older adult population, this significant and rapid weight loss may not be always beneficial. Even though there is a major fat mass reduction with body composition improvements, the lean mass loss, even though much lower than fat loss, can’t be overlooked in the frailest and sarcopenic population. Similarly to GLP-1 receptor agonists, gastrointestinal adverse effects may also lead to dehydration and pronounce malnutrition in fragile older adult population. More data on therapy in older adults and patients with sarcopenic obesity are needed to determine the role of tirzepatide in treatment.

#### Naltrexone/Bupropion

Naltrexone/bupropion is a fixed combination treatment option for management of overweight and obesity in addition to exercise and hypocaloric diet sold under names MYSIMBA (EU) and CONTRAVE (USA) [74]. It has been approved for use in 2014 by both the FDA and the EMA for adult patients with obesity defined as BMI > 30 kg/m^2^ or BMI > 27 kg/m^2^ with presence of one or more complications such as type 2 diabetes, dyslipidaemia or hypertension [75].

Naltrexone/bupropion is taken orally twice per day (total daily dose: 32 mg naltrexone hydrochloride and 360 mg bupropion hydrochloride) after a previous 4-week escalation dosing scheme starting from 8 mg/90 mg, with a weekly increase by 8 mg/90 mg. The treatment efficacy should be evaluated after 16 weeks of regular use.

Bupropion is a dopamine/norepinephrine reuptake inhibitor also approved for treatment of depression, seasonal affective disorder and for smoking cessation. Bupropion stimulates proopiomelanocortin (POMC) neurons in hypothalamus and affects pathways providing anorexigenic signals [76]. This effect is further potentiated and prolonged by naltrexone which is an opioid receptor antagonist used in opioid and alcohol dependence [76].

Treatment for 56 weeks by 32/360 mg of naltrexone/bupropion in the main large clinical trials (COR-I, COR-II and COR-BMOD) compared with placebo group resulted in 6.1 versus 1.3%, 6.4 versus 1.2%, and 9.3 versus 5.1% body weight loss [79]. Of the 3239 subjects who participated in clinical trials with CONTRAVE, 2% (62 subjects) were 65 years and older and at the same time no patients were older than 75 years [80]. The number of older adult participants was relatively low, therefore, geriatric outcomes were not analysed separately in the trials. Sufficient data regarding the use in older adults is therefore lacking and naltrexone/bupropion should be used with caution in older adults.

Bupropion and its metabolites are a strong in vivo CYP2D6 inhibitors [77] which may pose problems in older adult patients using wide range of drugs belonging to CYP2D6 substrates, such as analgesics (codeine, tramadol), antidepressants, beta blockers (metoprolol, bisoprolol), tamoxifen and many more. More than 72 different drugs have cytochrome CYP2D6 mentioned within their FDA-approved label as of 2020 [78].

Both bupropion and naltrexone are substantially eliminated via kidney, which affects their possibility of adverse reactions in patients with renal impairment that is frequent in the older adult group and even more in older adult patients with type 2 diabetes. Consequently, this drug should be used with caution in individuals over the age of 65 years and is not recommended for older adults aged 75 years and more. Monitoring of renal function is recommended. Older adult patients may be more sensitive to some of the central nervous system-related adverse effects such as dizziness and tremor [75]. Bupropion is contraindicated in patients with seizure disorder and other conditions predisposing to seizures such as severe head injury, severe stroke, brain tumour and more [81].

Naltrexone/bupropion treatment showed significant weigh loss effect, however, it was not tested in more detail in the older adult population. Adverse effects including dizziness, tremor and nausea may have detrimental impact on older adult patients; the drug interactions are not negligible as well and therefore the medication should be used very cautiously.

#### Orlistat

Orlistat (sold under the name XENICAL, ALLI) is a drug indicated for treatment of obesity combined with hypocaloric diet in patients with BMI > 30 kg/m^2^ or BMI > 28 kg/m^2^ associated with risk factors (EMA) or patients with BMI over 30 kg/m^2^, patients with a BMI greater than 27 kg/m^2^ and the presence of risk factors including hypertension, diabetes and dyslipidaemia or as treatment for reduction of the risk for weight regain after prior weight loss (FDA) [82, 83].

Orlistat is administered orally, with a recommended dose being one capsule containing 120 mg of orlistat taken with water immediately before, during or up to 1 h after the main meal containing fats, three times per day. The treatment works best in synergy with mildly hypocaloric diet that contains approximately 30% of calories derived from fat.

Orlistat is a saturated derivative of endogenous lipstatin isolated from *Streptomyces toxytricini*. It inhibits pancreatic and gastric lipases, which play an essential role in breaking down fats in the diet. Inactivation results in lowered triglyceride hydrolysis and consequently absorption of free fatty acids (FFAs) [82]. The main effect comes from the local action in the gut, and systemic exposure is minimal; 95–97% of the medication is excreted in faeces.

The most common adverse effect arising from the mechanism of action is steatorrhea, sometimes in association with abdominal discomfort and pain and diarrhoea; these symptoms can be managed by following the recommended dietary regimen (< 30% fat in the diet). Orlistat has an effect on absorption of both lipophilic drugs such as antiepileptics, amiodarone (reduced systemic exposure up to 25%) [84], cyclosporine, levothyroxine and antiretrovirals. Warfarin used in combination with orlistat may also result in prolonged prothrombin time and international normalised ratio (INR) due to reduced vitamin K absorption [85]. Unabsorbed fat also binds to calcium in the intestinal lumen, resulting in excessive oxalate, therefore increasing risk of oxalate nephropathy, nephrolithiasis and even acute kidney injury [86]. It also decreases absorption of lipophilic vitamins A, D, E and K and their supplementation is recommended in patients treated with orlistat.

All these effects should be considered in the older adult population who are often subjected to polypharmacy. However, data regarding use of orlistat in the older adult population are still lacking. Older adult population was not included in the XENDOS trial evaluating prevention of development of type 2 diabetes in obese patients by orlistat. The age eligibility criteria were 30–60 years of age, missing the population of interest of this article [87]. Efficacy of orlistat in XENDOS trials was 2.4% total body weight loss after 4 years [79]. In other trials, orlistat showed significant weight reduction 5.63% compared with 2.3% in placebo group [88]. Representation of the older adults was also low in other randomized controlled trials [89, 90], and data from this age group were not evaluated separately. There is no reason, however, to expect other adverse effects in the older adult population than the ones already known in younger patients. The main issues arise from increased fat presence in the stool and decreased absorption of vitamins, more precisely vitamin D, which alone is frequently deficient in the older adult population [91].

Orlistat has significant weight-reducing effect, however, data including older adult population are scarce or missing. Adverse gastrointestinal effects arising from the mechanism of action and the effect on drug absorption should be considered when prescribing this medication to older adults, especially those at risk of malnutrition and those using other prescription medication.

A summary of currently available pharmacotherapy of obesity in older adults is provided in the table below (Table 3).

**Table 3:** Currently available obesity pharmacotherapy

Drug	Class	Brand name	Administration	Maintenance dose	Main adverse effects	Trials included older adults
Liraglutide	GLP-1 agonist	Saxenda, Victoza	Subcutaneous injection	Up to 3.0 mg daily	Gastrointestinal disorders (nausea, diarrhoea, vomiting, constipation)	Yes, with comparable safety and efficacy profile to non-older adult group
Semaglutide	GLP-1 agonist	Ozempic, Wegovy, Rybelsus	Subcutaneous injection, oral	Up to 1mg weekly Up to 2.4mg weekly Up to 14mg daily	Gastrointestinal disorders (nausea, diarrhoea, vomiting, constipation)	Yes, with comparable safety and efficacy profile to non-older adult group
Tirzepatide	Dual GLP-1 and GIP agonist	Mounjaro, Zepbound	Subcutaneous injection	5, 10, and 15 mg weekly	Gastrointestinal disorders (nausea, diarrhoea, vomiting, constipation)	Yes, with comparable safety and efficacy profile to non-older adult group
Orlistat	Lipase inhibitor	Xenical, Alli	Oral	120 mg three times a day with main meals	Gastrointestinal disorders (diarrhoea, steatorrhea, abdominal pain, anal fissures)	Older adult population not included in trials
Naltrexone/bupropion	Opioid antagonist/ dopamine- norepinephrine- reuptake inhibitor	Mysimba, Contrave	Oral	32/360 mg divided into two daily doses	Gastrointestinal (nausea, constipation, vomiting), dizziness, dry mouth	2% of CONTRAVE trial participants were older adults, not enough data

## Sarcopenic Obesity and Related Therapy

Sarcopenia or sarcopenic obesity pose a specific challenge for the choice of optimal treatment strategy. Similar to obesity without sarcopenia, lifestyle modifications alongside with dietary changes protein, vitamin D, calcium and other micronutrient supplementation provide the cornerstone of treatment of sarcopenic obesity. The details are included in chapter 3.1.

To date, none of the abovementioned pharmacotherapies is indicated for specific treatment of sarcopenic obesity. There is no pharmacological intervention that provided solid evidence for treatment of sarcopenia [92]. Some conflicting data on muscle mass exist in GLP-1 receptor agonists group, however, more focus on sarcopenic obesity and body composition endpoints is necessary. Pronounced weight-reducing effect of these medications can be detrimental in frail patients with SO. Even though there is no tailored pharmacotherapy for SO, some interesting medications are being tested for both sarcopenia and sarcopenic obesity.

One of the most promising medications currently being evaluated is bimagrumab. Bimagrumab is a human monoclonal antibody that stimulates skeletal muscle growth by activin type II receptor (ActRII) inhibition. A 48-week phase 2 clinical trial led to 20.5% reduction of fat mass (compared with 0.5% in placebo group) and increased lean mass by 3.6% in contrast to − 0.8% with placebo (*p* < 0.001) [93, 94]. Other therapies targeting sarcopenia component including drugs involving renin–angiotensin system (RAS) or hormonal pathways are under evaluation. Further evidence is, however, needed and more detailed analysis is beyond the scope of this publication.

## Conclusions

Therapy of obesity in older adults should include mild calorie restriction and multimodal physical training including aerobic, balance, strength and flexibility exercises. Concomitant calcium, protein, vitamin D and other macro/micronutrient supplementation is necessary to reduce effects of weight loss on muscle mass and possible progression of sarcopenia and frailty. Pharmacotherapy should be chosen carefully with consideration to patient’s overall health status, comorbidities and other pharmacotherapy.

As of today, there are no officially recommended pharmacological treatment guidelines for older adult people with obesity. According to the Canadian Adult Obesity Clinical Practice Guidelines, pharmacotherapy should be used as an adjunct to medical nutrition therapy, physical activity and psychological interventions in obese adults. According to these guidelines, there are four medications indicated for long term treatment of obesity: liraglutide, semaglutide, naltrexone/bupropion and orlistat. These guidelines from 2022 state that pharmacotherapy for obesity management can be used for individuals with BMI ≥ 30 kg/m^2^ or BMI ≥ 27 kg/m^2^ with adiposity-related complications, in conjunction with medical nutrition therapy, physical activity and psychological interventions. The pharmacological regimens are as follows: semaglutide 2.4 mg weekly, liraglutide 3.0 mg daily, naltrexone/bupropion 16 mg/180 mg twice daily and orlistat 120 mg three times a day [95]. These guidelines, however, target the whole adult population without specific attention to older adults.

Selection of patients eligible for weight loss intervention should consider individual risk–benefit ratio and possible consequences of further lean mass reduction that can be observed across all treatment modalities. Weight loss should be recommended in older adult individuals with BMI of 30 kg/m^2^ and greater with weight-related comorbidity or functional limitations [35]. Clinicians should be aware of possible adverse effects arising from weight reduction, especially in frail patients, older adults with sarcopenic obesity or in cases of rapid uncontrolled weight loss.

Possible malnutrition, sarcopenia, loss of bone mineral density and resulting frailty should always be considered when deciding about weight reducing intervention. It is also important to keep in mind that every adverse effect arising from pharmacotherapy of obesity may aggravate concomitant medical conditions, remarkably in the context of malnutrition or dehydration commonly represented in older adult population.

## References

[1] Wang YC, McPherson K, Marsh T, Gortmaker SL, Brown M. Health and economic burden of the projected obesity trends in the USA and the UK. Lancet. 2011;378(9793):815–25. 21872750 10.1016/S0140-6736(11)60814-3

[2] Grimm W, Becker HF. Obesity, sleep apnea syndrome, and rhythmogenic risk. Herz. 2006;31(3):213–8 (**quiz 9**). 16770557 10.1007/s00059-006-2800-3

[3] Bastien M, Poirier P, Lemieux I, Despres JP. Overview of epidemiology and contribution of obesity to cardiovascular disease. Prog Cardiovasc Dis. 2014;56(4):369–81. 24438728 10.1016/j.pcad.2013.10.016

[4] Pati S, Irfan W, Jameel A, Ahmed S, Shahid RK. Obesity and cancer: a current overview of epidemiology, pathogenesis, outcomes, and management. Cancers (Basel). 2023;15(2):485. 36672434 10.3390/cancers15020485 PMC9857053

[5] Lega IC, Lipscombe LL. Review: diabetes, obesity, and cancer-pathophysiology and clinical implications. Endocr Rev. 2020;41(1):33–52. 10.1210/endrev/bnz014 31722374

[6] Preston SH, Stokes A. Contribution of obesity to international differences in life expectancy. Am J Public Health. 2011;101(11):2137–43. 21940912 10.2105/AJPH.2011.300219 PMC3222401

[7] Lung T, Jan S, Tan EJ, Killedar A, Hayes A. Impact of overweight, obesity and severe obesity on life expectancy of Australian adults. Int J Obes (Lond). 2019;43(4):782–9. 30283076 10.1038/s41366-018-0210-2

[8] Stephenson J, Smith CM, Kearns B, Haywood A, Bissell P. The association between obesity and quality of life: a retrospective analysis of a large-scale population-based cohort study. BMC Public Health. 2021;21(1):1990. 34732156 10.1186/s12889-021-12009-8 PMC8567540

[9] WHO WHO. Obesity. Feb. 21, 2022. https://www.who.int/health-topics/obesity. Accessed 9 Dec 2023.

[10] Purnell JQ. Definitions, classification, and epidemiology of obesity. In: Feingold KR, Anawalt B, Blackman MR, Boyce A, Chrousos G, Corpas E, et al., editors. Endotext. South Dartmouth, MA: MDText.com, Inc; 2000. 25905390

[11] Batsis JA, Villareal DT. Sarcopenic obesity in older adults: aetiology, epidemiology and treatment strategies. Nat Rev Endocrinol. 2018;14(9):513–37. 30065268 10.1038/s41574-018-0062-9 PMC6241236

[12] WHO. Waist circumference and waist-hip ratio: report of a WHO expert consultation. Geneva; 8–11 December 2008.

[13] (NAASO) NAAftSoO. Executive summary. Obes Res. 1998;6(S2):51S-210S.

[14] Ceniccola GD, Castro MG, Piovacari SMF, Horie LM, Corrêa FG, Barrere APN, et al. Current technologies in body composition assessment: advantages and disadvantages. Nutrition. 2019;62:25–31. 30826596 10.1016/j.nut.2018.11.028

[15] Winter JE, MacInnis RJ, Wattanapenpaiboon N, Nowson CA. BMI and all-cause mortality in older adults: a meta-analysis. Am J Clin Nutr. 2014;99(4):875–90. 24452240 10.3945/ajcn.113.068122

[16] Cruz-Jentoft AJ, Bahat G, Bauer J, Boirie Y, Bruyere O, Cederholm T, et al. Sarcopenia: revised European consensus on definition and diagnosis. Age Ageing. 2019;48(1):16–31. 30312372 10.1093/ageing/afy169 PMC6322506

[17] Malmstrom TK, Morley JE. SARC-F: a simple questionnaire to rapidly diagnose sarcopenia. J Am Med Dir Assoc. 2013;14(8):531–2. 23810110 10.1016/j.jamda.2013.05.018

[18] Donini LM, Busetto L, Bischoff SC, Cederholm T, Ballesteros-Pomar MD, Batsis JA, et al. Definition and diagnostic criteria for sarcopenic obesity: ESPEN and EASO consensus statement. Obes Facts. 2022;15(3):321–35. 35196654 10.1159/000521241 PMC9210010

[19] Batsis JA, Barre LK, Mackenzie TA, Pratt SI, Lopez-Jimenez F, Bartels SJ. Variation in the prevalence of sarcopenia and sarcopenic obesity in older adults associated with different research definitions: dual-energy X-ray absorptiometry data from the National Health and Nutrition Examination Survey 1999–2004. J Am Geriatr Soc. 2013;61(6):974–80. 23647372 10.1111/jgs.12260

[20] Yang M, Jiang J, Hao Q, Luo L, Dong B. Dynapenic obesity and lower extremity function in elderly adults. J Am Med Dir Assoc. 2015;16(1):31–6. 25227695 10.1016/j.jamda.2014.06.019

[21] Zibellini J, Seimon RV, Lee CM, Gibson AA, Hsu MS, Sainsbury A. Effect of diet-induced weight loss on muscle strength in adults with overweight or obesity—a systematic review and meta-analysis of clinical trials. Obes Rev. 2016;17(8):647–63. 27126087 10.1111/obr.12422

[22] Villareal DT, Chode S, Parimi N, Sinacore DR, Hilton T, Armamento-Villareal R, et al. Weight loss, exercise, or both and physical function in obese older adults. N Engl J Med. 2011;364(13):1218–29. 21449785 10.1056/NEJMoa1008234 PMC3114602

[23] Simati S, Kokkinos A, Dalamaga M, Argyrakopoulou G. Obesity paradox: fact or fiction? Curr Obes Rep. 2023;12(2):75–85. 36808566 10.1007/s13679-023-00497-1

[24] Wolk R, Bertolet M, Singh P, Brooks MM, Pratley RE, Frye RL, et al. Prognostic value of adipokines in predicting cardiovascular outcome: explaining the obesity paradox. Mayo Clin Proc. 2016;91(7):858–66. 27289411 10.1016/j.mayocp.2016.03.020 PMC4935584

[25] Bahat G, Kilic C, Ozkok S, Ozturk S, Karan MA. Associations of sarcopenic obesity versus sarcopenia alone with functionality. Clin Nutr. 2021;40(5):2851–9. 33940398 10.1016/j.clnu.2021.04.002

[26] Ozkok S, Aydin CO, Sacar DE, Catikkas NM, Erdogan T, Bozkurt ME, et al. Sarcopenic obesity versus sarcopenia alone with the use of probable sarcopenia definition for sarcopenia: associations with frailty and physical performance. Clin Nutr. 2022;41(11):2509–16. 36219979 10.1016/j.clnu.2022.09.005

[27] Bosello O, Vanzo A. Obesity paradox and aging. Eat Weight Disord. 2021;26(1):27–35. 31865598 10.1007/s40519-019-00815-4

[28] WHO. Obesity and overweight. 9 June 2021. https://www.who.int/news-room/fact-sheets/detail/obesity-and-overweight. Accessed 10 Dec 2023.

[29] National Center for Health Statistics. Health US. Normal weight, overweight, and obesity among adults aged 20 and over, by selected characteristics United States, selected years 1988–1994 through 2015–2018—Con. 2019. https://www.cdc.gov/nchs/data/hus/2019/026-508.pdf. Accessed 4 Dec 2023.

[30] Carl Baker HOCL. Obesity Statistics. 12 January 2023. https://researchbriefings.files.parliament.uk/documents/SN03336/SN03336.pdf. Accessed 10 Dec 2023.

[31] Malenfant JH, Batsis JA. Obesity in the geriatric population—a global health perspective. J Glob Health Rep. 2019;3:e2019045. 10.29392/joghr.3.e2019045 PMC8136402 34027129

[32] WHO WHO. Ageing and health. 1 October 2022. https://www.who.int/news-room/fact-sheets/detail/ageing-and-health. Accessed 9 Dec 2023.

[33] Volkert D, Beck AM, Cederholm T, Cruz-Jentoft A, Hooper L, Kiesswetter E, et al. ESPEN practical guideline: clinical nutrition and hydration in geriatrics. Clin Nutr. 2022;41(4):958–89. 35306388 10.1016/j.clnu.2022.01.024

[34] Waters DL, Ward AL, Villareal DT. Weight loss in obese adults 65years and older: a review of the controversy. Exp Gerontol. 2013;48(10):1054–61. 23403042 10.1016/j.exger.2013.02.005 PMC3714333

[35] Mathus-Vliegen EM, Obesity Management Task Force of the European Association for the Study of O. Prevalence, pathophysiology, health consequences and treatment options of obesity in the elderly: a guideline. Obes Facts. 2012;5(3):460–83. 10.1159/000341193 22797374

[36] Uchitomi R, Oyabu M, Kamei Y. Vitamin D and sarcopenia: potential of vitamin D supplementation in sarcopenia prevention and treatment. Nutrients. 2020;12(10):3189. 33086536 10.3390/nu12103189 PMC7603112

[37] Lee PG, Jackson EA, Richardson CR. Exercise prescriptions in older adults. Am Fam Physician. 2017;95(7):425–32. 28409595

[38] Wolfe BM, Kvach E, Eckel RH. Treatment of obesity: weight loss and bariatric surgery. Circ Res. 2016;118(11):1844–55. 27230645 10.1161/CIRCRESAHA.116.307591 PMC4888907

[39] Koliaki C, Liatis S, le Roux CW, Kokkinos A. The role of bariatric surgery to treat diabetes: current challenges and perspectives. BMC Endocr Disord. 2017;17(1):50. 28797248 10.1186/s12902-017-0202-6 PMC5553790

[40] English WJ, DeMaria EJ, Hutter MM, Kothari SN, Mattar SG, Brethauer SA, et al. American Society for Metabolic and Bariatric Surgery 2018 estimate of metabolic and bariatric procedures performed in the United States. Surg Obes Relat Dis. 2020;16(4):457–63. 32029370 10.1016/j.soard.2019.12.022

[41] Iranmanesh P, Boudreau V, Ramji K, Barlow K, Lovrics O, Anvari M. Outcomes of bariatric surgery in elderly patients: a registry-based cohort study with 3-year follow-up. Int J Obes (Lond). 2022;46(3):574–80. 34837011 10.1038/s41366-021-01031-w

[42] Mabeza RM, Mao Y, Maynard K, Lee C, Benharash P, Yetasook A. Bariatric surgery outcomes in geriatric patients: a contemporary, nationwide analysis. Surg Obes Relat Dis. 2022;18(8):1005–11. 35589528 10.1016/j.soard.2022.04.014

[43] Ladenheim EE. Liraglutide and obesity: a review of the data so far. Drug Des Devel Ther. 2015;9:1867–75. 25848222 10.2147/DDDT.S58459 PMC4386791

[44] Drucker DJ. GLP-1 physiology informs the pharmacotherapy of obesity. Mol Metab. 2022;57: 101351. 34626851 10.1016/j.molmet.2021.101351 PMC8859548

[45] Iepsen EW, Torekov SS, Holst JJ. Liraglutide for type 2 diabetes and obesity: a 2015 update. Expert Rev Cardiovasc Ther. 2015;13(7):753–67. 26106933 10.1586/14779072.2015.1054810

[46] EMA EMA. Saxenda—summary of product characteristics. 16/04/2015 12/09/2023. https://www.ema.europa.eu/en/documents/product-information/saxenda-epar-product-information_en.pdf. Accessed 8 Dec 2023.

[47] Pi-Sunyer X, Astrup A, Fujioka K, Greenway F, Halpern A, Krempf M, et al. A randomized, controlled trial of 3.0 mg of liraglutide in weight management. N Engl J Med. 2015;373(1):11–22. 26132939 10.1056/NEJMoa1411892

[48] Rubino DC, Raquel & Kahan, Scott & Kushner, Robert & Wilding, John & Jepsen, Cecilie & Smolarz, Gabriel & Wyatt, Holly. Age no Impediment to Effective Weight Loss with Liraglutide 3.0 Mg: Data from Two Randomized Trials. In: Nutrology IJo, editor. XXI I Congresso Brasileiro de Nutrologia; 2018.

[49] Perna S, Guido D, Bologna C, Solerte SB, Guerriero F, Isu A, et al. Liraglutide and obesity in elderly: efficacy in fat loss and safety in order to prevent sarcopenia. A perspective case series study. Aging Clin Exp Res. 2016;28(6):1251–7. 26749118 10.1007/s40520-015-0525-y

[50] Bahat G, Ozkok S. How to adjust muscle mass while defining sarcopenia component of sarcopenic obesity: is body weight sufficient enough to represent body size? Aging Clin Exp Res. 2023;35(3):723–4. 36622546 10.1007/s40520-022-02326-2

[51] Xiang J, Qin L, Zhong J, Xia N, Liang Y. GLP-1RA liraglutide and semaglutide improves obesity-induced muscle atrophy via SIRT1 pathway. Diabetes Metab Syndr Obes. 2023;16:2433–46. 37602204 10.2147/DMSO.S425642 PMC10439806

[52] Tonoike M, Chujo D, Noda M. Efficacy and safety of liraglutide added to insulin therapy in elderly patients with type 2 diabetes. Endocrinol Diabetes Metab. 2019;2(1): e00043. 30815572 10.1002/edm2.43 PMC6354751

[53] Gilbert MP, Bain SC, Franek E, Jodar-Gimeno E, Nauck MA, Pratley R, et al. Effect of liraglutide on cardiovascular outcomes in elderly patients: a post hoc analysis of a randomized controlled trial. Ann Intern Med. 2019;170(6):423–6. 30508430 10.7326/M18-1569

[54] Yoshida M, Yoshida A, Oh E, Yamamoto N, Sasaki E, Yoshida S, ET AL. 671-P: liraglutide possibly prevents progression of dementia in patients with type 2 diabetes. Diabetes. 2021;70(Supplement_1):671.

[55] HANSSEN R, RIGOUX L, KUZMANOVIC B, IGLESIAS S, KRETSCHMER AC, SCHLAMANN M, et al. Liraglutide restores impaired associative learning in individuals with obesity. Nat Metab. 2023;5(8):1352–63. 37592007 10.1038/s42255-023-00859-y PMC10447249

[56] EMA EMA. Ozempic—summary of product characteristics. 21/02/2018 03/05/2023. https://www.ema.europa.eu/en/documents/product-information/ozempic-epar-product-information_en.pdf. Accessed 8 Dec 2023.

[57] Warren MCL, Trachtenbarg D, Nayak G, Wijayasinghe N, Cariou B. Efficacy and safety of once-weekly semaglutide in elderly subjects with type 2 diabetes: post hoc analysis of SUSTAIN 1–5 trials. Diabetologia. 2017;60(Suppl 1):1–608.28795195

[58] Warren M, Chaykin L, Trachtenbarg D, Nayak G, Wijayasinghe N, Cariou B. Semaglutide as a therapeutic option for elderly patients with type 2 diabetes: pooled analysis of the SUSTAIN 1–5 trials. Diabetes Obes Metab. 2018;20(9):2291–7. 29687620 10.1111/dom.13331 PMC6099273

[59] Pratley RE, Aroda VR, Catarig AM, Lingvay I, Ludemann J, Yildirim E, et al. Impact of patient characteristics on efficacy and safety of once-weekly semaglutide versus dulaglutide: SUSTAIN 7 post hoc analyses. BMJ Open. 2020;10(11): e037883. 33199417 10.1136/bmjopen-2020-037883 PMC7670946

[60] Wilding JPH, Batterham RL, Calanna S, Davies M, Van Gaal LF, Lingvay I, et al. Once-weekly semaglutide in adults with overweight or obesity. N Engl J Med. 2021;384(11):989–1002. 33567185 10.1056/NEJMoa2032183

[61] Rubino DM, Greenway FL, Khalid U, O’Neil PM, Rosenstock J, Sorrig R, et al. Effect of weekly subcutaneous semaglutide vs daily liraglutide on body weight in adults with overweight or obesity without diabetes: the STEP 8 randomized clinical trial. JAMA. 2022;327(2):138–50. 35015037 10.1001/jama.2021.23619 PMC8753508

[62] Yamada Y, Yabe D, Hertz CL, Horio H, Nakamura J, Nielsen AM, et al. Efficacy and safety of oral semaglutide by baseline age in Japanese patients with type 2 diabetes: a subgroup analysis of the PIONEER 9 and 10 Japan trials. Diabetes Obes Metab. 2022;24(2):321–6. 34622548 10.1111/dom.14571 PMC9299616

[63] Uchiyama S, Sada Y, Mihara S, Sasaki Y, Sone M, Tanaka Y. Oral semaglutide induces loss of body fat mass without affecting muscle mass in patients with type 2 diabetes. J Clin Med Res. 2023;15(7):377–83. 37575352 10.14740/jocmr4987 PMC10416191

[64] Min T, Bain SC. The role of tirzepatide, dual GIP and GLP-1 receptor agonist, in the management of type 2 diabetes: the SURPASS clinical trials. Diabetes Ther. 2021;12(1):143–57. 33325008 10.1007/s13300-020-00981-0 PMC7843845

[65] FDA USFaDA. Zepbound—full prescribing information. 2022. https://www.accessdata.fda.gov/drugsatfda_docs/label/2023/217806s000lbl.pdf. Accessed 8 Dec 2023.

[66] EMA EMA. Mounjaro: summary of opinion (post authorisation). 2023. https://www.ema.europa.eu/en/documents/smop/chmp-post-authorisation-summary-positive-opinion-mounjaro-ii-07_en.pdf. Accessed 2 Jan 2024.

[67] FDA USFaDA. Mounjaro—full prescribing information. 2022. https://www.accessdata.fda.gov/drugsatfda_docs/label/2022/215866s000lbl.pdf. Accessed 8 Dec 2023.

[68] EMA EMA. Mounjaro—summary of product characteristics. 25/11/2022 26/10/2023. https://www.ema.europa.eu/en/documents/product-information/mounjaro-epar-product-information_en.pdf. Accessed 8 Dec 2023.

[69] Rosenstock J, Wysham C, Frias JP, Kaneko S, Lee CJ, Fernandez Lando L, et al. Efficacy and safety of a novel dual GIP and GLP-1 receptor agonist tirzepatide in patients with type 2 diabetes (SURPASS-1): a double-blind, randomised, phase 3 trial. Lancet. 2021;398(10295):143–55. 34186022 10.1016/S0140-6736(21)01324-6

[70] Abdalla MA, Soyiri I, Atkin S, Sathyapalan T. Tirzepatide as a novel therapeutic option for patients with type 2 diabetes: a pooled analysis of subgroups of SURPASS 1–5 trials. J Diabetol. 2023;14(2):65–73.

[71] Jastreboff AM, Aronne LJ, Ahmad NN, Wharton S, Connery L, Alves B, et al. Tirzepatide once weekly for the treatment of obesity. N Engl J Med. 2022;387(3):205–16. 35658024 10.1056/NEJMoa2206038

[72] Aronne L. ECO2023 NEWSLETTER: DAY THREE: new analysis shows improved body composition with tirzepatide is consistent across adult age groups with overweight or obesity. 2023. https://easo.org/eco2023-newsletter-day-three-new-analysis-shows-improved-body-composition-with-tirzepatide-is-consistent-across-adult-age-groups-with-overweight-or-obesity/. Accessed 1 Feb 2024.

[73] Kiyosue A, Dunn JP, Cui X, Hickey A, Hirase T, Imaoka T, et al. Safety and efficacy analyses across age and body mass index subgroups in East Asian participants with type 2 diabetes in the phase 3 tirzepatide studies (SURPASS programme). Diabetes Obes Metab. 2023;25(4):1056–67. 36545807 10.1111/dom.14952

[74] Onakpoya IJ, Lee JJ, Mahtani KR, Aronson JK, Heneghan CJ. Naltrexone-bupropion (Mysimba) in management of obesity: a systematic review and meta-analysis of unpublished clinical study reports. Br J Clin Pharmacol. 2020;86(4):646–67. 31918448 10.1111/bcp.14210 PMC7098870

[75] EMA EMA. Mysimba—summary of product characteristics. 15/04/2015. https://www.ema.europa.eu/en/documents/product-information/mysimba-epar-product-information_en.pdf. Accessed 4 Jan 2024.

[76] Kim KK. Understanding the mechanism of action and clinical implications of anti-obesity drugs recently approved in Korea. Korean J Fam Med. 2019;40(2):63–71. 30929417 10.4082/kjfm.19.0013 PMC6444089

[77] Costa R, Oliveira NG, Dinis-Oliveira RJ. Pharmacokinetic and pharmacodynamic of bupropion: integrative overview of relevant clinical and forensic aspects. Drug Metab Rev. 2019;51(3):293–313. 31124380 10.1080/03602532.2019.1620763

[78] Taylor C, Crosby I, Yip V, Maguire P, Pirmohamed M, Turner RM. A review of the important role of CYP2D6 in pharmacogenomics. Genes (Basel). 2020;11(11):1295. 33143137 10.3390/genes11111295 PMC7692531

[79] Tak YJ, Lee SY. Long-term efficacy and safety of anti-obesity treatment: where do we stand? Curr Obes Rep. 2021;10(1):14–30. 33410104 10.1007/s13679-020-00422-w PMC7787121

[80] FDA USFaDA. Contrave—full prescribing information. 2014. https://www.accessdata.fda.gov/drugsatfda_docs/label/2014/200063s000lbl.pdf. Accessed 10 Dec 2023.

[81] Huecker MR, Smiley A, Saadabadi A. Bupropion. Treasure Island: StatPearls; 2024. 29262173

[82] Bansal AB, Al Khalili Y. Orlistat. StatPearls. Treasure Island (FL) ineligible companies. Disclosure: Yasir Al Khalili declares no relevant financial relationships with ineligible companies. 2024.

[83] EMA EMA. Xenical—summary of product characteristics. 2009. https://www.ema.europa.eu/en/documents/product-information/xenical-epar-product-information_en.pdf. Accessed 10 Dec 2023.

[84] Zhi J, Moore R, Kanitra L, Mulligan TE. Effects of orlistat, a lipase inhibitor, on the pharmacokinetics of three highly lipophilic drugs (amiodarone, fluoxetine, and simvastatin) in healthy volunteers. J Clin Pharmacol. 2003;43(4):428–35. 12723464 10.1177/0091270003252236

[85] MacWalter RS, Fraser HW, Armstrong KM. Orlistat enhances warfarin effect. Ann Pharmacother. 2003;37(4):510–2. 12659605 10.1345/aph.1C122

[86] Humayun Y, Ball KC, Lewin JR, Lerant AA, Fulop T. Acute oxalate nephropathy associated with orlistat. J Nephropathol. 2016;5(2):79–83. 27152294 10.15171/jnp.2016.14 PMC4844913

[87] Torgerson JS, Hauptman J, Boldrin MN, Sjostrom L. XENical in the prevention of diabetes in obese subjects (XENDOS) study: a randomized study of orlistat as an adjunct to lifestyle changes for the prevention of type 2 diabetes in obese patients. Diabetes Care. 2004;27(1):155–61. 14693982 10.2337/diacare.27.1.155

[88] Jain SS, Ramanand SJ, Ramanand JB, Akat PB, Patwardhan MH, Joshi SR. Evaluation of efficacy and safety of orlistat in obese patients. Indian J Endocrinol Metab. 2011;15(2):99–104. 21731866 10.4103/2230-8210.81938 PMC3125014

[89] Finer N, James WP, Kopelman PG, Lean ME, Williams G. One-year treatment of obesity: a randomized, double-blind, placebo-controlled, multicentre study of orlistat, a gastrointestinal lipase inhibitor. Int J Obes Relat Metab Disord. 2000;24(3):306–13. 10757623 10.1038/sj.ijo.0801128

[90] Davidson MH, Hauptman J, DiGirolamo M, Foreyt JP, Halsted CH, Heber D, et al. Weight control and risk factor reduction in obese subjects treated for 2 years with orlistat: a randomized controlled trial. JAMA. 1999;281(3):235–42. 9918478 10.1001/jama.281.3.235

[91] Kweder H, Eidi H. Vitamin D deficiency in elderly: risk factors and drugs impact on vitamin D status. Avicenna J Med. 2018;8(4):139–46. 30319955 10.4103/ajm.AJM_20_18 PMC6178567

[92] Bahat G, Ozkok S. The current landscape of pharmacotherapies for sarcopenia. Drugs Aging. 2024;41(2):83–112. 38315328 10.1007/s40266-023-01093-7

[93] Melson E, Ashraf U, Papamargaritis D, Davies MJ. What is the pipeline for future medications for obesity? Int J Obes (Lond). 2024. 10.1038/s41366-024-01473-y. 10.1038/s41366-024-01473-y PMC11971045 38302593

[94] Heymsfield SB, Coleman LA, Miller R, Rooks DS, Laurent D, Petricoul O, et al. Effect of bimagrumab vs placebo on body fat mass among adults with type 2 diabetes and obesity: a phase 2 randomized clinical trial. JAMA Netw Open. 2021;4(1): e2033457. 33439265 10.1001/jamanetworkopen.2020.33457 PMC7807292

[95] Pedersen SD MP, Wharton S. Canadian Adult Obesity Clinical Practice Guidelines: Pharmacotherapy for Obesity Management. 2022. https://obesitycanada.ca/guidelines/pharmacotherapy/. Accessed 7 Dec 2023.

